# Diagnostic Value of Plasma Long Non-coding SLC26A4 Antisense RNA 1 Combined with Magnetic Resonance Imaging in Rectal Cancer

**DOI:** 10.5152/tjg.2024.23558

**Published:** 2024-12-01

**Authors:** Zhiqian Li, Mei Pu, Peng Zhou, Tao Zhang, Yang Xu, Yusui Zhang

**Affiliations:** Department of Radiology, First Affiliated Hospital of Guizhou University of Traditional Chinese Medicine, Guiyang, China

**Keywords:** Rectal cancer, lncRNA SLC26A4-AS1, miR-3174, MRI, diagnosis

## Abstract

**Background/Aims:**

The prevalence of rectal cancer is increasing every year due to changes in living and eating habits. Early diagnosis contributes to the treatment and survival of patients. This study investigated the feasibility of employing SLC26A4-AS1 combined with magnetic resonance imaging (MRI) for diagnosing rectal cancer.

**Materials and Methods:**

The current study involved 125 patients with rectal cancer and an equal number of healthy individuals. The study focused on assessing the relationship between SLC26A4-AS1 expression and clinical data among patients with rectal cancer by analyzing the expression levels. MRI blood perfusion parameters (K^trans^, K_ep_, V_e_, and incremental area under the curve (iAUC)) were measured in the patients with rectal cancer. The regulation of SLC26A4-AS1 on the biological function of rectal cancer cells was analyzed by Cell Counting Kit-8 (CCK-8) method, flow cytometry, and Transwell assay. Furthermore, luciferase activity assays and RNA-binding protein immunoprecipitation assay (RIP) were conducted to elucidate the relationship between SLC26A4-AS1 and microRNA-3174 (miR-3174).

**Results:**

A significant reduction in SLC26A4-AS1 expression was observed in rectal cancer alongside a significant increase in miR-3174 levels. SLC26A4*-*AS1 expression was negatively correlated with K^trans^ and K_ep_ values, but not with V_e_ or iAUC values. Cell experiments confirmed the inhibitory effect of SLC26A4-AS1 overexpression on the growth of rectal cancer cells. Additionally, SLC26A4-AS1 sponged miR-3174 mediated the progression of rectal cancer. The enriched miR-3174 may counteract the suppression of the biological activity of oe-SLC26A4-AS1 on rectal cancer cells.

**Conclusion:**

: SLC26A4-AS1 may serve as a diagnostic tool for rectal cancer, mediating tumor progression by directly targeting miR-3174.

**Main Points**

- SLC26A4-AS1 expression in rectal cancer was significantly reduced, while miR-3174 levels were markedly increased.
- SLC26A4-AS1 expression was negatively correlated with Ktrans and Kep values, but not with Ve or iAUC values.
- SLC26A4-AS1 mediated the proliferation, apoptosis, migration, and invasion levels of rectal cancer cells through sponging miR-3174.
- SLC26A4-AS1 may be a novel diagnostic marker for rectal cancer.

## Introduction

Rectal cancer, a prevalent digestive tract tumor primarily located near the anus, affects over 700 000 individuals worldwide annually, as per the 2020 statistics.^1,2^ In China, shifts in lifestyle and social settings have led to a steady rise in the incidence and fatalities related to rectal cancer.^3^ The etiology of rectal cancer is relatively complex and may be the result of a combination of factors, such as dietary habits and environmental factors.^4^ While surgical interventions can significantly control rectal cancer progression, the survival outcome for many patients in the advanced stage remains suboptimal and the recurrence rate is high. Therefore, early identification of high-risk groups prone to recurrence holds significant practical importance for rectal cancer prevention and treatment outcome enhancement.

Typically, endoscopy and MRI serve as common diagnostic and preoperative assessment tools for rectal cancer.^5-7^ Basendowah et al. have proposed that MRI has high confidence in the diagnosis and treatment allocation of patients with rectal cancer.^5^ While widely endorsed diagnostic methods exist, recent literature has increasingly highlighted the role of lncRNAs in tumor prevention and regulation by modulating miRNAs.^8^ For instance, lncRNAs have been shown to have diagnostic, prognostic, and therapeutic potential in liver diseases, including hepatocellular carcinoma.^9^ SLC26A4-AS1, located on human chromosome 7q22.3, has been identified as a potential therapeutic target for thyroid cancer, breast cancer, and glioma,^10-12^ but its mechanism of action in rectal cancer has not been elucidated. Meanwhile, SLC26A4-AS1 is thought to act as a cancer suppressor in tumors.^13^ Notably, miR-3174 was reported to have an oncogenic role in different tumors, including rectal cancer.^14,15^ The bioinformatics site predicted the presence of binding sites between SLC26A4-AS1 and miR-3174, prompting speculation about their potential efficacy in diagnosing and treating rectal cancer.

The current study aims to evaluate the mechanism of SLC26A4-AS1 in rectal cancer, assessing its potential as a diagnostic biomarker. Additionally, it seeks to offer a theoretical framework for exploring the potential clinical value and pathological mechanism of SLC26A4-AS1 in rectal cancer.

## Materials and Methods

### Patient Inclusion and Sample Handling

The study enrolled individuals treated at First Affiliated Hospital of Guizhou University of Traditional Chinese Medicine hospital between March 2021 and August 2023. The participant pool comprised 125 individuals diagnosed with rectal cancer and 125 healthy individuals who underwent physical examinations. Among the patients with rectal cancer, those who underwent MRI and received confirmation from specialists were included, while the following cases were excluded: 1) patients with multiple diseases; 2) individuals who were unable to participate due to personal or external factors; 3) patients who underwent long-term tumor treatment. The control group consisted of healthy individuals who received regular physical examinations with no abnormal results. The study was conducted with the permission of the Ethics Committee of First Affiliated Hospital of Guizhou University of Traditional Chinese Medicine hospital (approval no: K2020-002, date: 10 December 2020), and all participants provided written informed consent.

The obtained blood from the participants was stored in anticoagulant tubes, repeatedly inverted and thoroughly mixed, and then processed by centrifugation (4°C, 1200 g, 10 min) in a centrifuge (Eppendorf, Germany). The resultant plasma samples were stored in a refrigerator at −80°C.

### MRI Examination

Patients with rectal cancer underwent MRI scans using a 1.5T MRI scanner (Siemens, Germany). DWI images with b = 800 s/mm^2^ were selected by 3 radiologists in a blind method to describe the region of interest (ROI) of the tumor lesions (avoiding the cystic degeneration and necrosis areas), and the solid plane measurement was performed. Subsequently, the collected data included and analyzed the K^trans^, K_ep_, V_e_, and iAUC values. In addition, control healthy individuals did not undergo MRI examination.

K^trans^ and K_ep_ refer to the transport constants of contrast agents between plasma and blood vessels, where K^trans^ is related to blood flow and permeability, and K_ep_ reflects vascular permeability. V_e_ is the volume fraction of the contrast agent permeating from the plasma into the extravascular cellular space, and the iAUC value is expressed as the initial area under the curve of the contrast agent concentration. The above parameters effectively reflect the physiological characteristics of the patient’s blood vessels, which in turn predict the progression of rectal cancer.

### Cell Lines and Transfection

Human rectal cancer cells SNU-61 and SNU-283 were purchased from the Korean Cell Line Bank, and American Type Culture Collection (ATCC) supplied rectal cancer cells SW1463 and SW837, as well as control colon epithelial cells FHC. All these cell lines were cultured in a 6-well cell culture plate with the addition of RPMI 1640 medium and statically in a CO_2_ cell incubator at 37°C.

The overexpression vector plasmid pcDNA3.1 was derived from GenePharma Co., LTD. (Shanghai, China), and the negative control (oe-NC) and overexpressed SLC26A4-AS1 (oe-SLC26A4-AS1) vectors were synthesized. Upon reaching the logarithmic growth phase, the cells were transfected using Lipofectamine 3000 (Invitrogen, USA) and incubated for 48 h.

### Detection of RNA Expression

TRIzol reagent (1 mL; Sigma-Aldrich, USA) and chloroform reagent (200 μL) were added to the rectal cancer plasma sample (200 μL). The mixture was thoroughly mixed in centrifuge tubes and then centrifuged to obtain the supernatant. An equal volume of isopropanol solution was added for mixing, and RNA samples were obtained. Next, cDNA was synthesized using the Precision nanoScript2 Reverse Transcription Kit (Primer Design, UK). Subsequently, the quantitative reverse transcription PCR (RT-qPCR) system was configured by sequentially adding the relevant reagents from SYBR qPCR Green Master Mix (Vazyme, China) with cDNA as the reaction template, and assessed using the 7500 Real-Time PCR system (Applied Biosystems, USA). The endogenous control of SLC26A4-AS1 and miR-3174 was performed by GAPDH^16^ and U6, and the RNA levels were quantified using the 2^-ΔΔCt^ method.

### CCK-8 Cell Proliferation Assay

The proliferation ability of SW1463 and SNU-283 cells was tested using the CCK-8 Kit (Dojindo). Transfected SW1463 or SNU-283 cells were seeded into 96-well plates (4 × 10^3^ cells/well). CCK-8 reagent was added at intervals of 0, 24, 48, and 72 h. Finally, the absorbance at 450 nm was measured for each well.

### Annexin V Cell Apoptosis Assay

Transfected SW1463 and SNU-283 cells were cultured in 6-well plates and washed with phosphate buffered saline (PBS) solution after reaching the logarithmic stage. The cells underwent treatment with a FITC Annexin V cell apoptosis assay kit (BD Biosciences, USA), and the apoptosis levels were assessed using flow cytometry (Becton Dickinson, USA).

### Transwell Assay

The experimental procedures of the Transwell cell migration and invasion assay are similar, both of which are performed in a 24-well chamber. The specific steps are as follows: (1) RPMI-1640 medium for suspending transfected cells was added into the upper side (2 × 10^4^ pieces/well), and 10% FBS and RPMI-1640 medium were transferred into the underside; (2) After 24 h of induction, the cells that migrated below were fixed and stained; (3) Cell counting was performed under the microscope (Philips, Netherlands). Importantly, Matrigel coating of the upper chamber is necessary for invasion assays.

### Luciferase Reporter Assay

Wild-type SLC26A4-AS1 (WT) and mutant-type SLC26A4-AS1 (MUT) vectors were constructed using pmirGLO (Promega, China). Following this, a co-transfection of mimic-NC or miR-3174 mimic, along with Lipofectamine 3000 reagents, was performed on SNU-283 cells, which were cultured in 24-well plates. Subsequently, the luciferase activity of SNU-283 cells was measured.

### RIP Assay

SNU-283 cells underwent lysis and processing in accordance with the Magna RIP RNA binding protein kit (Millipore, USA). Then, Ago2 and IgG antibodies were introduced. The IgG antibody was regarded as the negative control, and the levels of SLC26A4-AS1 and miR-3174 were detected by RT-qPCR.

### Statistical Analysis

All statistical data were processed with SPSS version 20 (IBM SPSS Corp.; Armonk, NY, USA) and GraphPad 10 software (GraphPad Software,San Diego, CA, USA), and the results are presented as the mean ± standard deviation. The chi-square test was used to evaluate the correlation between clinical features and abnormal expression of SLC26A4-AS1 in patients with rectal cancer. The relationship between MRI perfusion parameters and SLC26A4-AS1 was determined using the Pearson method. Differences among various groups were assessed using Student’s *t*-test or one-way analysis of variance (ANOVA) with post-hoc testing. The receiver operating characteristic (ROC) curve was constructed to evaluate the potential diagnostic value of SLC26A4-AS1 in rectal cancer. Each set of experiments was replicated at least thrice to ensure result accuracy, and statistical significance was set at *P* < .05.

## Results

### SLC26A4-AS1 was Downregulated in Rectal Cancer

**Figure 1.**
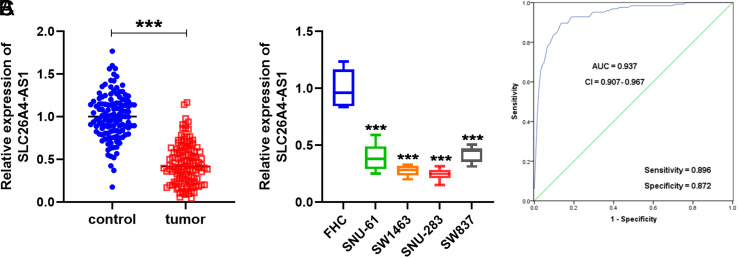
Expression and diagnostic significance of SLC26A4-AS1. (A) Determination of SLC26A4-AS1 in plasma of rectal cancer and healthy human plasma. (B) Levels of SLC26A4-AS1 in rectal cancer cells (SNU-61, SW1463, SNU-283, SW837) and normal cell FHC were detected by RT-qPCR. (***P < .001). (C) The diagnostic potential of SLC26A4-AS1 in rectal cancer was measured by the ROC curve (AUC = 0.937).

Quantitative reverse transcription PCR (RT-qPCR) was used to quantify the levels of SLC26A4-AS1 in rectal cancer plasma and cells. Figure 1A illustrates the observed downregulation of plasma SLC26A4-AS1 in patients with rectal cancer compared to healthy controls. SLC26A4-AS1 expression was decreased in the included rectal cancer cells compared with normal cells FHC and aberrantly decreased in SW1463 and SNU-283 cells (Figure 1B). Based on this, SW1463 and SNU-283 cells were used for later investigations.

### Clinical Indicators and Diagnostic Factors of Patients

Table 1 outlines the correlation between SLC26A4-AS1 expression and clinical indicators among the included patients with rectal cancer. Referring to the average expression, the patients were categorized into 2 groups: SLC26A4-AS1 low level (n = 67) and SLC26A4-AS1 high level (n = 58). Aberrant SLC26A4-AS1 expression showed close associations with the TNM (tumor-node-metastasis) stage (*P* = .021), lymphatic metastasis (*P* = .025), and distant metastases (*P* = .039) in patients with rectal cancer. Moreover, through ROC curve analysis, the area under the curve was determined as 0.937 (CI = 0.907-0.967), with sensitivity and specificity values of 0.896 and 0.872, as presented in Figure 1C. 

### Correlation Between MRI Blood Perfusion Parameters and SLC26A4-AS1

**Figure 2.**
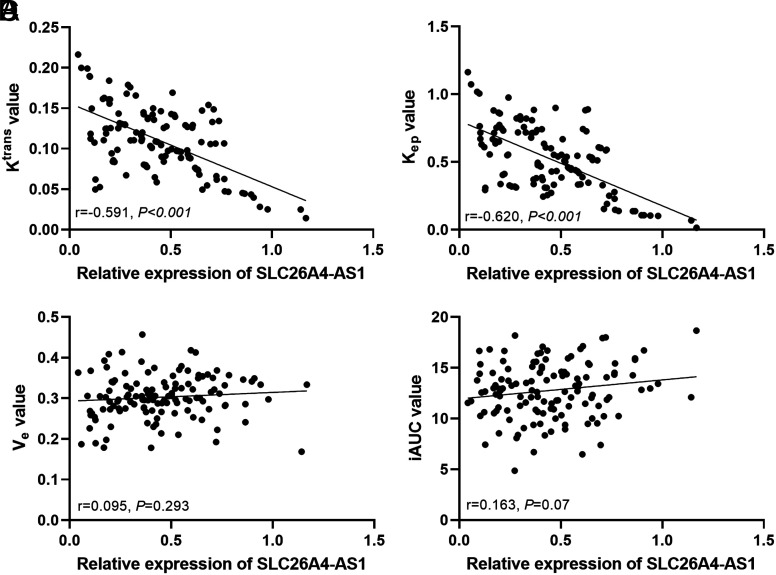
Correlation analysis between MRI blood perfusion parameters and SLC26A4-AS1 level. (A) Correlation between K^trans^ value and SLC26A4-AS1 (*r* = −0.591, *P* < .001). (B) Correlation between K_ep_ value and SLC26A4-AS1 (*r* = −0.620, *P* < .001). (C) Correlation between V_e_ value and SLC26A4-AS1 (*P* > .05). (D) Correlation between iAUC value and SLC26A4-AS1 (*P* > .05).

The Pearson method was employed to reveal the relationship between MRI blood perfusion parameters and SLC26A4-AS1 expression. Figure 2A depicted a decrease in the K^trans^ value alongside an increase in SLC26A4-AS1, highlighting a negative correlation (*r* = −0.591, *P* < .001). Similarly, Figure 2B indicated that the K_ep_ value was also inversely proportional to the SLC26A4-AS1 level (*r* =− 0.620, *P* < .001). However, no correlation was observed between V_e_ and iAUC and SLC26A4-AS1 expression in Figures 2C and  D (*P* > .05).

### oe-SLC26A4-AS1 Suppresses Rectal Cancer Cellular Behaviors

**Figure 3.**
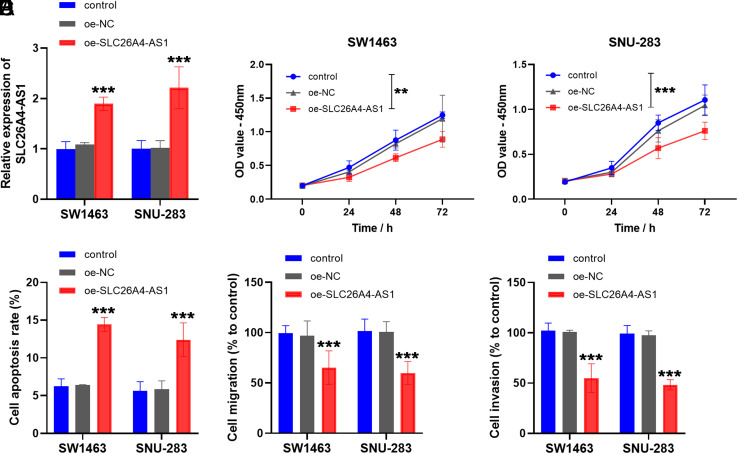
Effect of up-regulated SLC26A4-AS1 on rectal cancer cells. (A) Transfection efficiency of oe-SLC26A4-AS1 in SW1463 and SNU-283 cells. (B) and (C) SW1463 and SNU-283 cell viability was measured by the CCK-8 method. (D) Apoptosis rate of rectal cancer cells after transfection with oe-SLC26A4-AS1. (E) Migratory level of the cells was represented. (F) Invasive ability of cells was measured. (**P < .01, ***P < .001).

In the cell assay, overexpression of SLC26A4-AS1 was transfected into SW1463 and SNU-283 cells, leading to an elevation in SLC26A4-AS1 within the oe-SLC26A4-AS1 group compared to the control group as confirmed by RT-qPCR (Figure 3A). Compared to the control group, the proliferation ability of SW1463 and SNU-283 cells was inhibited after oe-SLC26A4-AS1 transfection (Figures 3B and  C). Moreover, as displayed in Figure 3D, apoptosis rates of SW1463 and SNU-283 cells were significantly increased in the oe-SLC26A4-AS1 group compared to the control group. The Transwell assays depicted a decrease in cell migration (Figure 3E **)** and cell invasion **(** Figure 3F **)** upon transfection with oe-SLC26A4-AS1.

### Targeting the Relationship Between SLC26A4-AS1 and miR-3174

**Figure 4.**
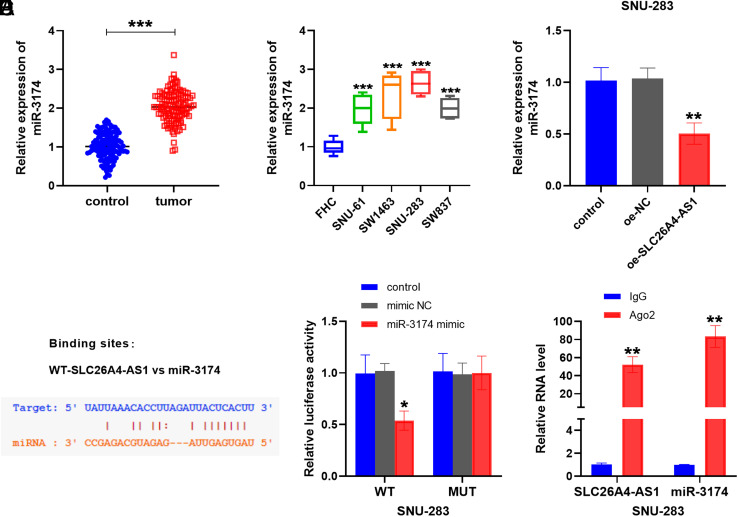
Interaction of SLC26A4-AS1 and miR-3174. (A) Compared with plasma miR-3174 of healthy subjects, miR-3174 was up-regulated in rectal cancer plasma. (B) The level of miR-3174 was increased in rectal cancer cells. (C) Overexpression of SLC26A4-AS1 inhibited the miR-3174 level in SNU-283 cells. (D) The binding sites between SLC26A4-AS1 and miR-3174. (E) Luciferase activity was examined in SNU-283 cells co-transfected with miR-3174 mimic or mimic NC and WT-SLC26A4-AS1 or MUT-SLC26A4-AS1. (F) Ago2 antibody significantly up-regulated the SLC26A4-AS1 and miR-3174 expression. (*P < .05, **P < 0.01, ***P < .001).

RT-qPCR analysis was conducted to assess miR-3174 expression in plasma and cell samples of rectal cancer, revealing an increase in miR-3174 levels compared to controls (Figures 4A and  B). The overexpression of SLC26A4-AS1 suppressed the miR-3174 level in SNU-283 cells** (** Figure 4C **)**. Further, an online database suggested the presence of binding sites between SLC26A4-AS1 and miR-3174, as presented in Figure 4D. In accordance with the findings displayed in Figure 4E, the introduction of the miR-3174 mimic resulted in reduced luciferase activity of WT-SLC26A4-AS1. Additionally, Figure 4F highlights the active expression of SLC26A4-AS1 and miR-3174 within the Ago2 group compared to the IgG group.

### MiR-3174 Mimic Remedied the Suppression of Cell Activity by oe-SLC26A4-AS1

**Figure 5.**
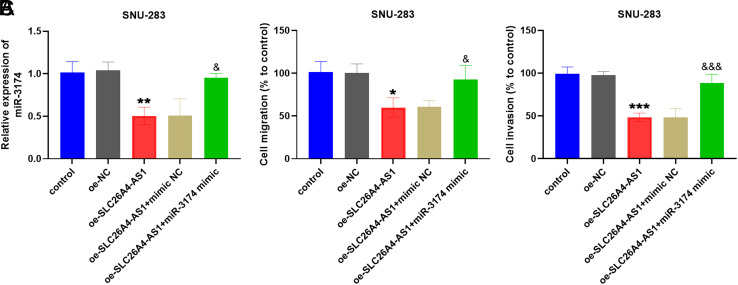
Effects of the miR-3174 mimic on SNU-28 cells. (A) Content of miR-3174 in SNU-28 cells transfected with oe-SLC26A4-AS1+miR-3174 mimic. (B) and (C) After transfection with oe-SLC26A4-AS1+miR-3174 mimic, the motility of SNU-28 cells was restored. (*P < .05, **P < .01, ***P < .001, with control; &*P* < .05, &&&*P* < .001, with oe-SLC26A4-AS1).

oe-SLC26A4-AS1 and miR-3174 mimic were co-transfected into SNU-283 cells to confirm the involvement of miR-3174 in SLC26A4-AS1 regulation. It was observed in Figure 5A that miR-3174 content was upregulated in SNU-283 cells after transfection with oe-SLC26A4-AS1 + miR-3174 mimic, compared with after transfection of oe-SLC26A4-AS1. Furthermore, findings from Transwell assays reflected that the introduction of the miR-3174 mimic alleviated the inhibitory effect of oe-SLC26A4-AS1 on the migration ability of SNU-283 cells compared with the oe-SLC26A4-AS1 group (Figure 5B). Similarly, the cell invasion levels were reversed due to the increased presence of miR-3174 (Figure 5C).

## Discussion

Rectal cancer is a lethal tumor known for its tendency to metastasize easily and recur frequently.^17^ This study emphasized the investigation of diagnostic markers specific to patients with rectal cancer to enable timely monitoring for potential lesions and early detection and disease diagnosis.

In reported studies related to SLC26A4-AS1, Han et al. observed reduced SLC26A4-AS1 expression in thyroid cancer. Their study demonstrated that SLC26A4-AS1 mediates tumor metastasis by regulating DDX5.^10^ Yi et al. reported diminished SLC26A4-AS1 levels, closely linked to prognosis, survival, and immune infiltration in patients with breast cancer.^11^ Through RT-qPCR detection, we observed that SLC26A4-AS1 also showed low levels in plasma and cell samples of rectal cancer compared with healthy controls, which was consistent with the above conclusions. Concurrently, analysis of clinical indicators revealed the association between abnormally low SLC26A4-AS1 expression and TNM stage, lymphatic metastasis, and distant metastasis of rectal cancer patients, implying that SLC26A4-AS1 may be involved in the process of rectal cancer. Furthermore, establishing ROC curves aided in elucidating the potential clinical diagnostic value of lncRNA.^18^ In earlier discussions on colorectal cancer, ROC analysis confirmed the ability of SNHG11 to distinguish early-stage patients.^19^ Similarly, our study concluded that SLC26A4-AS1 had an AUC of 0.937 in the identification of rectal cancer patients with high sensitivity and specificity in diagnosing rectal cancer, thereby holding promise as a diagnostic factor. 

Significantly, MRI stands as the primary modality for diagnosing and evaluating rectal cancer.^20,21^ The collection of MRI blood perfusion parameters can reflect the physiological characteristics of patients’ blood vessels, which is helpful in analyzing the process of rectal cancer. This study demonstrated that SLC26A4-AS1 expression was negatively correlated with K^trans^ and K_ep_ values, but not obviously correlated with V_e_ or iAUC. Literature suggests that elevated K^trans^ values accompany the deterioration of rectal cancer, while increased K_ep_ content is associated with angiogenesis in rectal cancer.^22^ Li et al. also underscored the diagnostic efficacy of MRI blood perfusion parameters for patients with rectal cancer.^23^ Therefore, the association of SLC26A4-AS1 with K^trans^ and K_ep_ values further elucidated the predictive and pathological staging potential of SLC26A4-AS1 in rectal cancer.

We explored the regulatory mechanism of SLC26A4-AS1 in rectal cancer cells through in vitro cell experiments. Upon transfection and overexpression of SLC26A4-AS1, suppression in the growth and metastatic potential of rectal cancer cells was observed, coupled with an increase in apoptosis levels, compared to the control group. This led us to hypothesize that elevated SLC26A4-AS1 might influence rectal cancer progression by regulating the cell cycle. Recent studies have reported a decrease in the content of lncRNA RMST in colorectal cancer, with the upregulation of RMST exerting a negative effect on cell colony formation and triggering apoptosis,^24^ which was similar to our experimental results. Additionally, our bioinformatics predictions and luciferase activity assays showed that miR-3174 may be the downstream action site of SLC26A4-AS1. miR-3174, recognized as a candidate for tumor therapy, has exhibited aberrant expression in glioblastoma, endometrial cancer, and bladder cancer.^25-27^ Wang and colleagues confirmed that miR-3174 was upregulated in hepatocellular carcinoma, highlighting its role in mediating cell activities by binding to FOXO1.^15^ In this study, miR-3174 was assessed to be elevated in plasma and cells of rectal cancer compared to the healthy group and negatively regulated by SLC26A4-AS1. Cell recovery assays explained that transfection with miR-3174 mimic effectively counteracted the inhibitory effect of oe-SLC26A4-AS1 on the migration and invasion levels of rectal cancer cells. This indicates that SLC26A4-AS1 directly targets miR-3174 expression and mediates the progression of rectal cancer. It was also mentioned that the targeting of PCBD2 factor by miR-3174 may play an oncogenic role in the development of rectal cancer,^14^ which prompted our subsequent consideration of the downstream targets of miR-3174. In addition, exploring lncRNA sponge miRNA-mediated drug resistance in rectal cancer offers innovative treatment avenues.^28^ For example, the LINC00461/miR-593-5p/CCND1 regulatory network may regulate cisplatin resistance in rectal cancer.^29^ Recent reports also mentioned the function of lncRNAs in colorectal cancer by modulating JAK/STAT signaling.^30^ Additionally, the prognostic significance of lncRNA DLGAP1-AS2 in regulating the Trim21/ELOA/LHPP network in colorectal cancer provided a new perspective for the treatment of the tumor.^31^

Nonetheless, the limited number of plasma samples in this study inevitably reduces the robustness of our findings, necessitating a larger cohort of rectal cancer samples to substantiate our conclusions. Furthermore, supplementing our study with mouse experiments may more vividly reflect the clinical situation and enhance the credibility of our findings.

In conclusion, SLC26A4-AS1 was down-regulated in rectal cancer, which regulated tumor progression by directly sponging miR-3174 on cell growth and cycle. Enriched miR-3174 repaired the inhibitory function of silencing SLC26A4-AS1 on rectal cancer cells. SLC26A4-AS1 holds promise as a potential biomarker for diagnosing rectal cancer.

## Availability of Data and Materials

The data that support the findings of this study are available on request from the corresponding author.

## Figures and Tables

**Table 1.** Correlation between SLC26A4-AS1 expression and clinical indicators in patients with rectal cancer

Parameters	SLC26A4-AS1 Low level	SLC26A4-AS1 High level	*P*
N = 125	n = 67	n = 58	
Age			.743
≤ 60	32	26	
> 60	35	32	
Sex			.842
Male	37	31	
Female	30	27	
Tumor size			.062
≤ 2 cm	34	39	
> 2 cm	33	19	
TNM stage			.021
T1-T2	39	45	
T3-T4	28	13	
Lymphatic metastasis			.025
No	45	49	
Yes	22	9	
Distant metastases			.039
No	49	51	
Yes	18	7	
Differentiation			.363
Well-Moderate	35	35	
Poor	32	23	
